# Supplementary figures and images for: The Mutationathon highlights the importance of reaching standardization in estimates of pedigree-based germline mutation rates
Source: eLife. 2022 Jan 12;11:e73577. doi: 10.7554/eLife.73577 (PMC8830884; doi:10.7554/eLife.73577)

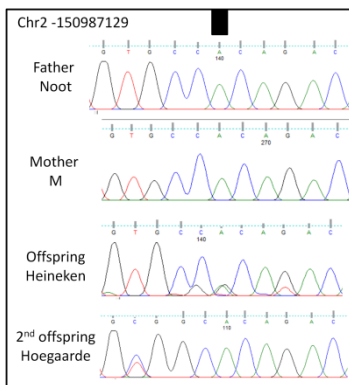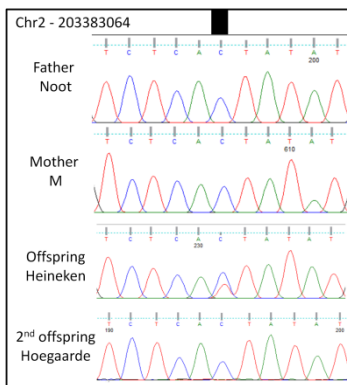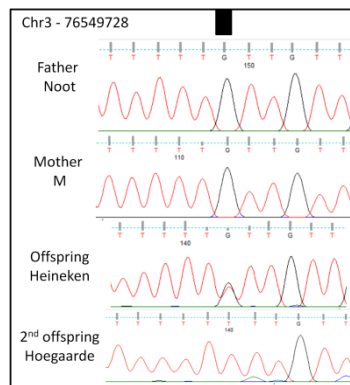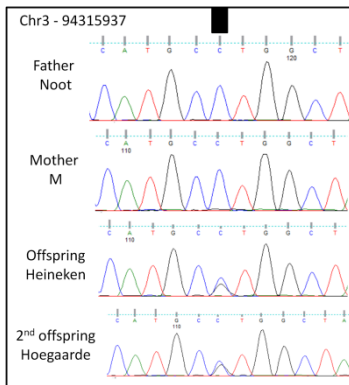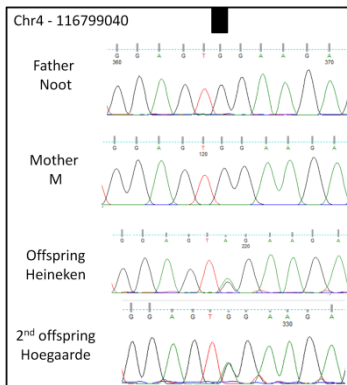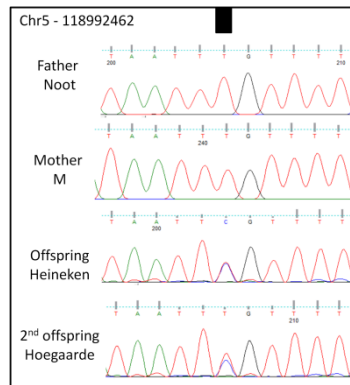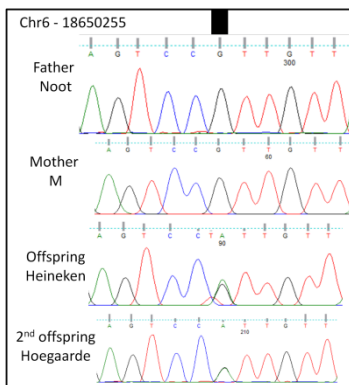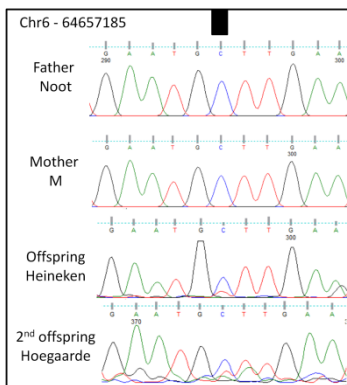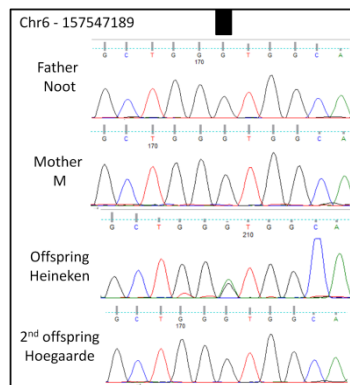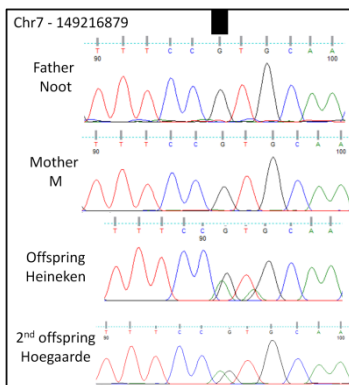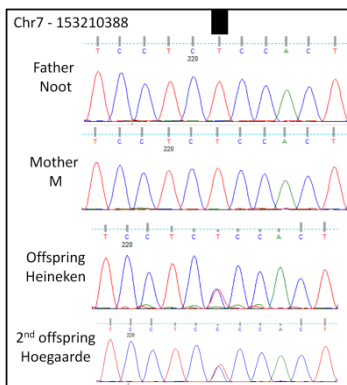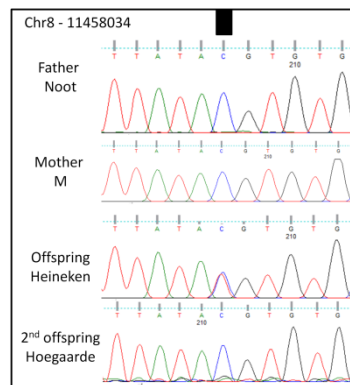

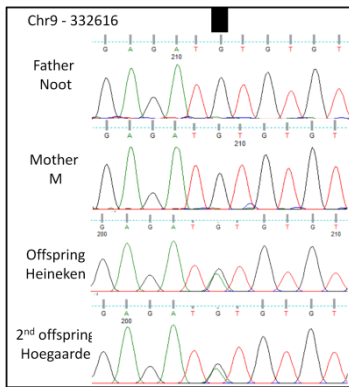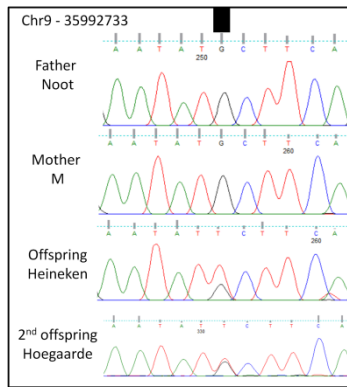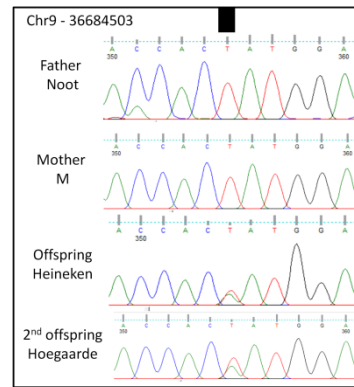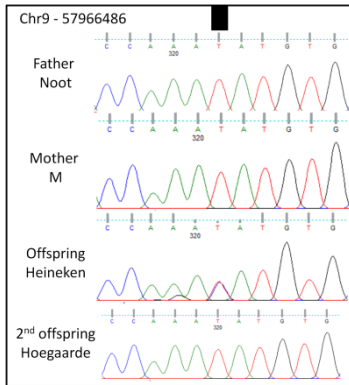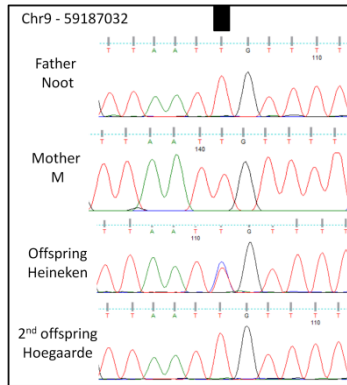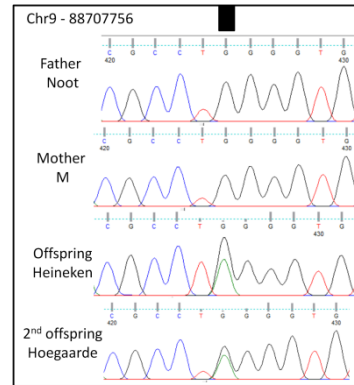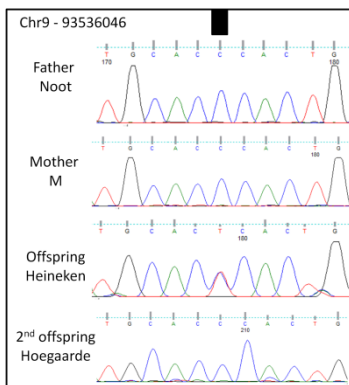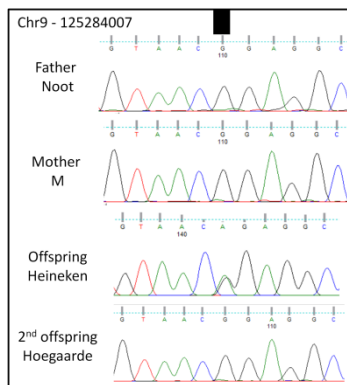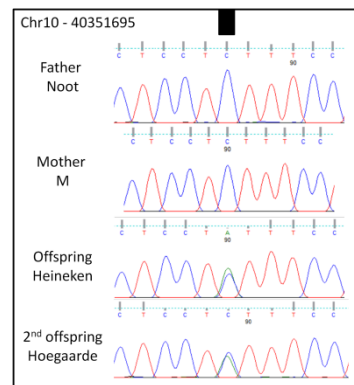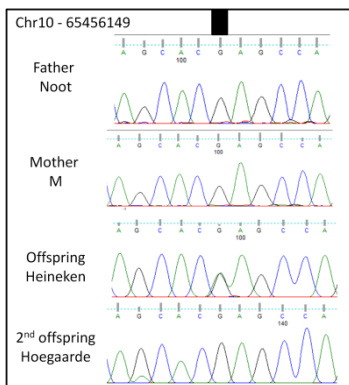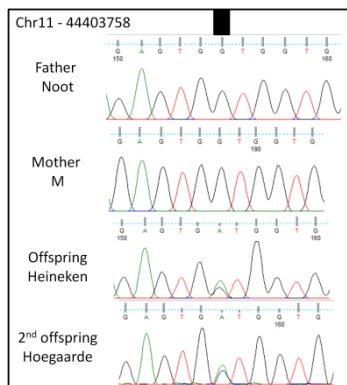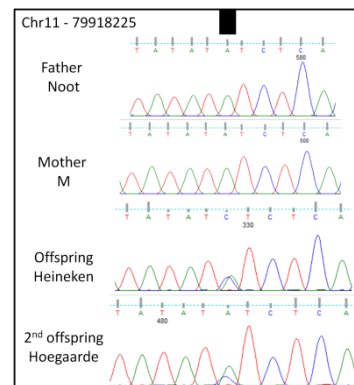

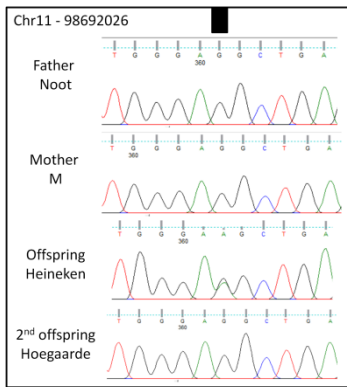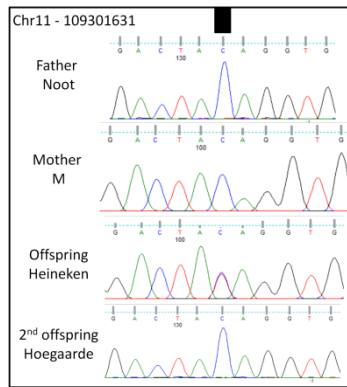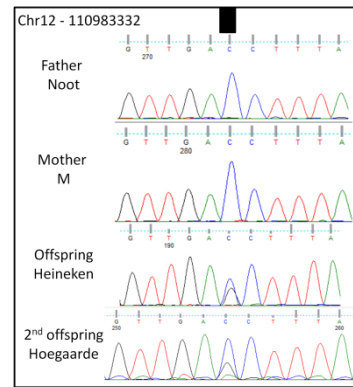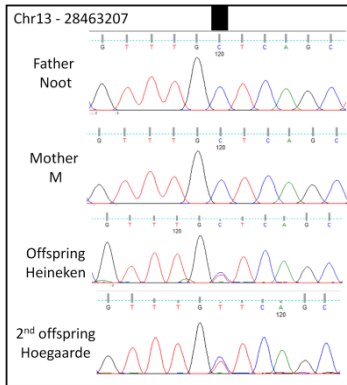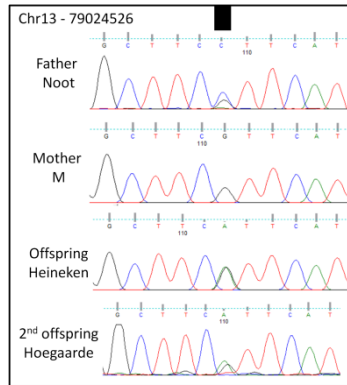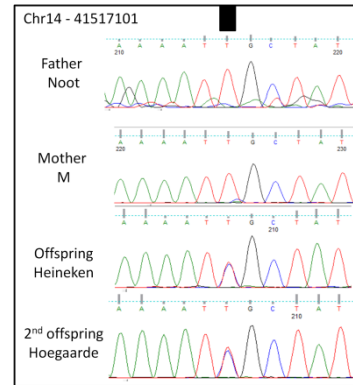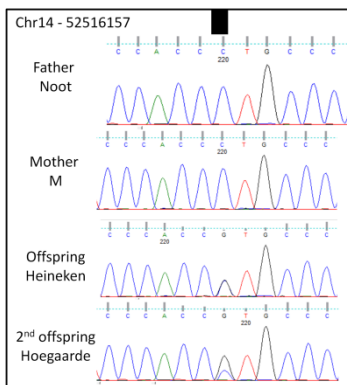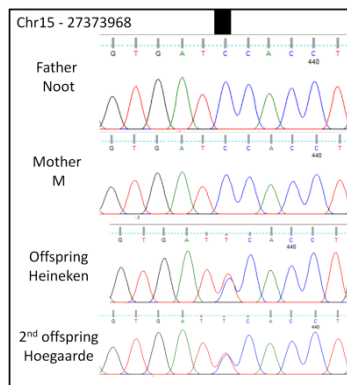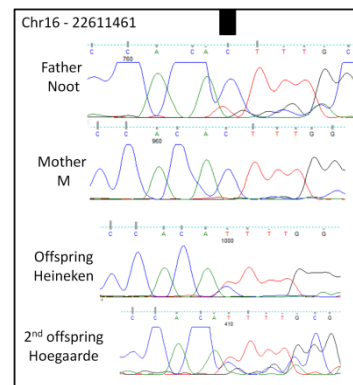

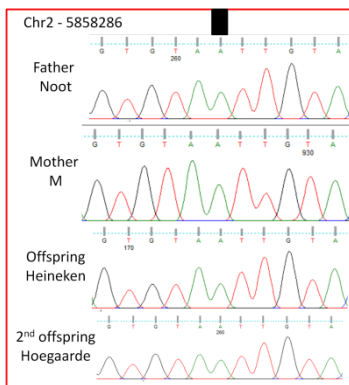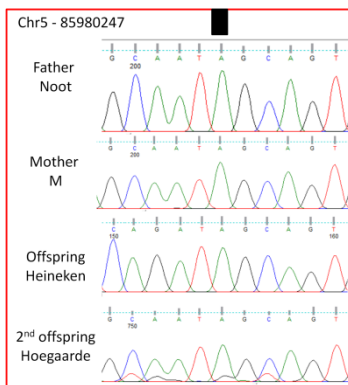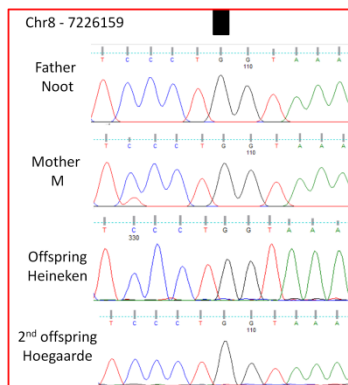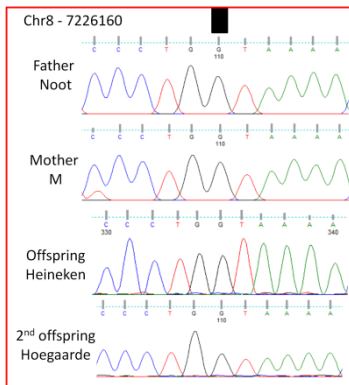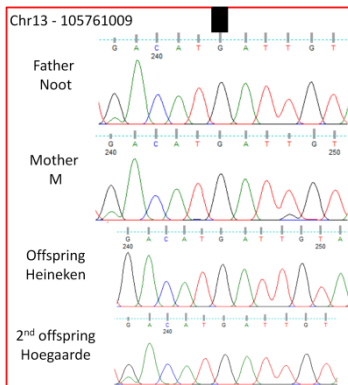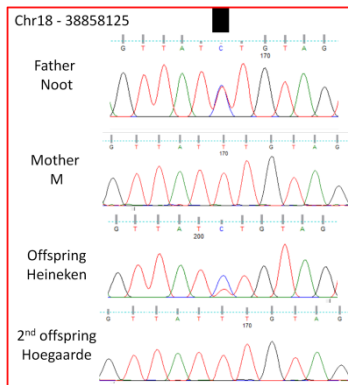

Supplement: Figure 3—source data 2. — For each alignment, the candidate germline mutation position is located under the black square. The last six chromatograms (surrounded by red boxes) are the candidates that were detected as false-positive candidates. [file elife-73577-fig3-data2.pdf]
