# Supplementary material for: The Mutationathon highlights the importance of reaching standardization in estimates of pedigree-based germline mutation rates
Source: eLife. 2022 Jan 12;11:e73577. doi: 10.7554/eLife.73577 (PMC8830884; doi:10.7554/eLife.73577)
Supplement: Supplementary file 1. [file elife-73577-supp1.docx]

**Supplementary file 1a – Study design and methodology of the studies on pedigree-based germline mutation rate estimation**. Information on the species studied, the number of trios, type of library preparation, the sequencing machine, sequencing depth, software used for mapping, variant calling, and filters used to detect the DNMs and calculate mutation rates are reported when available. Only recent studies from 2016 onwards are included.

| **Study** | **Species** | **Trios** | **Library** | **Sequencing** | **Depth** | **Mapping** | **Remove duplicates** | **BQSR** | **Variant calling** | **DNMs detection** | **Method FDR** | **FDR** | **Method FNR** | **FNR** | **Method *CG*** | ***CG*** | ***μ* per generation** |
| --- | --- | --- | --- | --- | --- | --- | --- | --- | --- | --- | --- | --- | --- | --- | --- | --- | --- |
| **Rahbari et al. 2016** | Human | 13 |  |  | 24.7X |  |  |  |  | DeNovoGear | Illumina resequencing and manual curation |  |  |  | Remove sites not passing the DP filter, in low complex region, not validated by primers, or filtered by DeNovoGear priors filters | 83.1% | Poisson distribution |
| **Smeds et al. 2016** | Collared flycatcher | 7 |  | Illumina HiSeq | 40X | BWA 0.7.5a | Picard MarkDuplicates | Yes | GATK 3.3.0 - HaplotypeCaller and GenotypeGVCFs | Customized filtering | Manual curation | 35% |  | 0 |  | 80% |  |
| **Wong et al. 2016** | Human | 719 |  | Complete Genomics Inc. and 61 also with Illumina | 60X | Complete Genomics’ Assembly (CGA) Pipeline versions 2.0.0–2.0.4 |  |  | CGA or Strelka and GATK | Customized filter and PhaseByTransmission | Overlap between different pipeline and different sequencing | 13% | Overlap between different pipeline and different sequencing | 25% | Remove from the commnly callable region the tandem repeats, duplication regions, alignability smaller than 1 | 72% | $\frac{nb_{de novo}\times SP \div SE}{2 \times CG}$  SP : specificity  SE : sensitivity |
| **Feng et al. 2017** | Herring | 12 | PCR free | Illumina HiSeq 2500 | 65.8X | BWA 0.6.2 |  |  | GATK 3.3.0 - HaplotypeCaller and Samtools 1.19 mpilup | Customized filtering, overlapping of variant caller | Sanger sequencing |  | Simulation and detection of true heterozygotes | 5.9% | Remove site with mappability lower than 1 and repeat region | 51% | $\frac{nb_{de novo all trios}}{2 \times nb_{trios}\times CG\times(1-FDR)}$ |
| **Harland et al. 2017** | Cattle | 5 | PCR free | Illumina HiSeq 2000 | 23X | BWA mem 0.7.9a-r786 | Picard MarkDuplicates | Yes | GATK 3.4 - HaplotypeCaller | Customized filters | Illumina resequencing | 3% |  |  | Sites passing the DP filters |  |  |
| **Jónsson et al. 2017** | Human | 1550 | PCR free and non  PCR free | Illumina | 35X | BWA mem 0.7.10 | Picard MarkDuplicates 1.117 |  | GATK- UnifiedGenotyper | Customized filtering | Discordance monozygotic twin | 3% | Simultation of SNPs | 3.86 % | Sites with DP > 10 X and < 120 X (only reads MQ > 20, windows of 10,000 bp) | 88% | $\frac{nb_{de novo}}{2 \times CG}$ |
| **Maretty et al. 2017** | Human | 150 |  | Illumina HiSeq 2000 | 78X | BWA mem 0.7.5a | yes | Yes | GATK - HaplotypeCaller | Customized filtering |  |  |  |  | 2.5 Gb | 87% |  |
| **Milholland et al. 2017** | Mouse | 8 | PCR free | Illumina HiSeq 2500 | 28.5X | BWA mem | Samtools | Yes | GATK - Unifiedgenotyper | DeNovoGear and VarScan2, kept overlapping candidates | Validation Sanger sequencing | 25% |  |  | Number of base callable | 57.4% | $\frac{nb_{de novo} \times(1-FDR)}{2 \times CG}$ |
| **Pfeifer 2017** | African green monkey | 3 |  | Illumina HiSeq 2000 | 22X | BWA mem 0.7.13 | Picard MarkDuplicates 2.1.1 | Yes | GATK 3.5 - HaplotypeCaller | Customized filtering | Manual curation | 91% | Mutations simulation | 15.6 % | Removed sites with DP_parent_ < 10, LowQual, MQ < 60, in repetitive regions, or incomplete genotyping | 57% | $\frac{nb_{de novo}}{2 \times CG \times(1-FNR)}$ |
| **Tatsumoto et al. 2017** | Chimpanzee | 1 | PCR free | Illumina HiSeq 2000 | 150X | BWA mem 0.6.1 | Picard MarkDuplicates 1.93 | Yes | GATK 2.1.9 - UnifiedGenotyper | Customized filtering | Sanger validation | 20% | DeNovoGear and validation | 0 | Remove sites with DP < filter | 45% | $\frac{nb_{de novo} \times(1-FDR)}{2 \times CG}$ |
| **Turner et al. 2017** | Human | 516 | PCR free | Illumina HiSeq X | 34.8X | BWA mem 0.7.8 | Picard MarkDuplicates 1.83 | Yes | GATK 3.5 - HaplotypeCaller and FreeBayes 1.0.1 | Customized filters and overlapping between variant caller | resequencing Sanger validation | 3.7% |  |  |  |  |  |
| **Malinski et al. 2018** | Cichlid (3 species) | 9 |  | Illumina HiSeq 2000 | 40X | BWA mem 0.7.10 | Picard MarkDuplicates |  | GATK 3.3.0 - HaplotypeCaller and samtools/bcftools 1.18 | Customized filters and overlapping between variant caller | From the binomial distribution of the AB | 5% | Read Position or Base Quality rank-sum test | 7.17% | Using GATK filters on non variant site | 91.5% | $\frac{nb_{de novo}}{2 \times CG}$ |
| **Martin et al. 2018** | Platypus | 2 |  | Illumina HiSeq 2000 | 16X | Stampy | Picard MarkDuplicates |  | PLATYPUS | PLATYPUS *de novo* variant caller |  |  |  |  |  | 46% | $\frac{nb_{de novo 1}+ nb_{de novo 2}}{CG_{trio 1}+CG_{trio 2}}$ |
| **Thomas et al. 2018** | Owl monkey | 14 | PCR free | Illumina HiSeq X | 37X | BWA mem 0.7.12 | Picard Mark Duplicates 1.105 |  | GATK 3.3.0 - HaplotypeCaller | Customized filtering | Strict filters | 0 | correction for AB filter | 44% |  |  | $\frac{nb_{de novo}}{2 \times CG \times(1-FNR)}$ |
| **Besenbacher et al. 2019** | Chimpanzee orangutan gorilla | 7,1,2 | PCR free | Illumina HiSeq X | 42X | BWA mem |  | Yes | GATK 3.8 - HaplotypeCaller | Customized filtering | Strict filters | 0 | Correction for sites filters |  | Probability of being a certain genotype given the depth | 32 - 79 % | $\frac{nb_{de novo}}{2 \times CG \times(1-FNR)}$ |
| **Koch et al. 2019** | Wolf | 4 |  | Illumina HiSeq 2000 | 23X | BWA 0.7.12 | GATK 3.5.0 | Yes | GATK 3.5.0 - UnifiedGenotyper | Customized filtering |  |  | FNR as a probability | 11.2 % | Filters on a subset of sites |  | Poisson distribution |
| **Lindsay et al. 2019** | Mouse | 15 |  | Illumina HiSeq | 25X |  |  |  | Bcftools and samtools | DeNovoGear 0.5 | Illumina resequencing |  |  |  | Site passing DP filter and DeNovoGear prior filters | 89.9% |  |
| **Sasani et al. 2019** | Human | 593 | NonPCR free | Illumina HiSeq X | 30X | BWA mem 0.7.15 | Samblaster | Yes | GATK 3.5.0 | Customized filters | WGS resequenging higher depth and transmission | 4.5% | "Missed heterozygote" also with transmition | 0.4% | mosdepth to calculate DP and remove lower than 12, remove low MQ < 20, remove low complexity regions | 83.3% | $\times\frac{1-FDR}{1-MHR}$ |
| **Kessler et al. 2020** | Human | 1449 | PCR free | Illumina HiSeq X | 38X | BWA mem |  | Yes | GotCloud pipeline | Customized filter and TrioDeNovo | call variant with TrioDeNovo | 1.8% | Used similar FNR than Jonsson et al | 3.86 % | Site passing DP and quality threshold |  | $\times\frac{1-FDR}{1-MHR}$ |
| **Wang et al. 2020** | Rhesus macaque | 14 | PCR free | Illumina HiSeq X | 40X | BWA mem 0.7.12 | Picard MarkDuplicates |  | GATK 3.6 | Customized filtering |  | 0 | Probability | 20% | Pass depth and homozygote reference filter | 88% | $\frac{nb_{de novo}}{2 \times CG \times(1-FNR)}$ |
| **Wu et al. 2020** | Baboon | 12 | PCR free | Illumina HiSeq X and Illumina HiSeq 2500 | 45X | BWA mem 0.7.9a | Samblaster 0.1.24 Picard MarkDuplicates 2.9.0 | Yes | GATK 3.7 - HaplotypeCaller and GenotypeGVCFs | Customized filtering | Binomial probability of transmission | 18% | Mutations simulation | 7.93 % | GATK CallableLoci tool (on depth) + remove if Alt in parents |  | Per sex likelihood |
| **Bergeron et al. 2021** | Rhesus macaque | 19 | NonPCR free | BGIseq500 | 81X | BWA mem 0.7.15 | Picard MarkDuplicates 2.7.1 |  | GATK 4.0.7.0 - HaplotypeCaller BP_resolution | Customized filtering | Manual curation | 11% | Correction for sites filters and AB filter | 4.28 % | Sites that pass HomRef, DP, and GQ filter | 89% | $\frac{nb_{de novo}\times(1-FDR)}{2 \times CG \times(1-FNR)}$ |
| **Campbell et al. 2021** | Grey mouse lemur | 2 |  | 10X genomics | 34.5X | 10x Genomic’s LongRanger v2.2.1 |  |  | GATK 3.8 within LongRanger | DeNovoGear v1.1.1 and VarScan2 v2.4.3, kept overlapping candidates | Error rate from technical replicates | 4.8 % | Error rate from technical replicates | 28% | Simulation with BAMsurgeon v1.0.0 | 84% | $\frac{nb_{de novo}}{2 \times CG}$ |
| **Wang et al. 2021** | Cat | 11 | PCR free | Illumina HiSeq X | 41X | BWA mem 0.7.12-r1039 | Picard MarkDuplicates 1.105 |  | GATK 3.6 - HaplotypeCaller | Customized filters |  |  | On the AB filter |  | Probability and correction for FNR | 72% | $\frac{nb_{de novo}}{2 \times CG \times(1-FNR)}$ |
| **Yang et al. 2021** | Marmoset | 1 | NonPCR free | Offspring 10X genomics and parent Illumina | Offspring 40X, Parents 75X | BWA ALN (v.0.7.12) | Picard MarkDuplicates |  | GATK 4.0.7.0 - HaplotypeCaller BP_resolution | Customized filtering | Validation with the other haplotype (maternal and paternal) |  | Correction for sites filters and AB filter |  | Sites that pass HomRef, DP, GQ, and AD filter | 41% | $\frac{nb_{de novo M}+ nb_{de novo F}}{CG_{M}\left( 1-FNR_{M} \right)+ CG_{F}(1-FNR_{F})}$  M : mother  F : father |

**Supplementary file 1b – Description of site filters included in the GATK Best Practices hard-filtering recommendations.** See GATK <https://gatk.broadinstitute.org/hc/en-us/articles/360035890471-Hard-filtering-germline-short-variants> for more details

| **Site filter** | **Explanation** |
| --- | --- |
| **QualByDepth (QD)** | The variant confidence (QUAL field) divided by the unfiltered depth of non-HomRef samples. Used to select high-quality calls, and correct for the depth differences between samples. It is advised by GATK to use QD instead of the depth or the QUAL directly. |
| **RMSMappingQuality (MQ)** | Root mean square of the mapping quality over all the reads at the site. Good mapping qualities are around 60. Using the root square includes the standard deviation. High standard deviations mean that reads are highly divergent from the mean in this location. |
| **FisherStrand (FS)** | The Phred-scaled probability of strand bias at the site. Used to remove sites where the alternative allele is only seen in either forward or reverse strands. FS = 0 means no bias. |
| **StrandOddsRatio (SOR)** | FS penalizes variants at the end of exons, thus, SOR is another way to estimate and correct for strand bias. |
| **MappingQualityRankSumTest (MQRankSum)** | Compares the mapping qualities of the reads with the reference allele with those of the alternate allele and corrects for any bias. Positive values indicate a better mapping quality for alternative alleles, negative values indicate a bias toward the mapping quality of reference allele, and 0 means no bias. |
| **ReadPosRankSumTest (ReadPosRankSum)** | Compares the positions of the reference and alternate alleles on the reads. Negatives values indicate that alternative alleles are at the end of reads more often than reference alleles. Positive values indicate the opposite while 0 indicates no position bias. |

**Supplementary file 1c – Several of the customized individual filters applied in the detection of germline mutations in the different reviewed studies.**

| **Studies** | **Variant calling** | ***Site filters (GATK recommended filters: QD <* 2.0 ; *MQ <* 40*.*0; *FS >* 60*.*0; *SOR >* 3*.*0; *MQRankSum<* -12.5; *ReadPosRankSum* < -8.0)** | **Depth** | **Genotype quality** | **Allelic depth parents** | **Allelic balance** |
| --- | --- | --- | --- | --- | --- | --- |
| **Smeds et al. 2016** | GATK 3.3.0 - HaplotypeCaller and GenotypeGVCFs |  | DPmin none DPmax none | GQ < 30 | AD > 0 | AB < 0.25 |
| **Harland et al. 2017** | GATK HaplotypeCaller (version 3.4) |  | DPmin 10 DPmax 60 | GQ < 40 (offspring) | AD > 0 |  |
| **Jónsson et al. 2017** | GATK- UnifiedGenotyper | “GATK best practice” | DPmin 12 DPmax none | GQ < 20 | AD > 1 | AB < 0.25 AB > 0.75 |
| **Maretty et al. 2017** | GATK - HaplotypeCaller (gvcf) | “GATK best practice” | DPmin 20 DPmax 150 | GQ_Hom_ < 80 GQ_Het_ < 250 | AD > 4 | AB < 0.3 |
| **Pfeifer 2017** | GATK 3.5 - HaplotypeCaller and GenotypeGVCFs | LowQual, MQ < 60 | DP_parent_ < 10 | None | AD > 0 | AB < 0.2 |
| **Tatsumoto et al. 2017** | GATK 2.1.9 - UnifiedGenotyper |  | DPmin av_i_ − 3σ DPmax av_i_ + 3σ | GQ_Hom_ < 100 GQ_Het_ < 200 | None | At least 1 Read alt forward and 1 alt reverse |
| **Thomas et al. 2018** | GATK 3.3.0 - HaplotypeCaller | QD < 2.0; MQ < 40.0; FS > 60.0; **~~SOR > 3.0~~**; MQRankSum < -12.5; ReadPosRankSum < -8.0 | DPmin 20 DPmax 60 | None | None | AB < 0.4 AB > 0.6 |
| **Besenbacher et al. 2019** | GATK 3.8 - HaplotypeCaller | FS > 20; ReadPosRankSum < -6 and > 6; BaseQualityRankSum; MappingQualityRankSum < 6 | DPmin 10 DPmax 1.9 × av_i_ | GQ < 65 | AD > 0 lowQ AD2 > 1 | AB < 0.3 |
| **Koch et al. 2019** | GATK 3.5.0 - UnifiedGenotyper | QD < 2.0; MQ < 40.0; FS > 60.0; **~~SOR > 3.0~~**; MQRankSum < -12.5; **ReadPosRankSum < 15** | DPmin 10 DPmax 100 | None | AD > 0 | At least 1 read with Alt |
| **Sasani et al. 2019** | GATK v3.5.0 |  | DPmin 12 DPmax none | GQ < 20 | AD > 0 | More than 0.3% of the reads for the 3rd generation (transmission was not possible) |
| **Wang et al. 2020** | GATK 3.6 | QD < 2.0; MQ < 40.0; FS > 60.0; **~~SOR > 3.0~~**; MQRankSum < -12.5; ReadPosRankSum < -8.0 | DPmin 20 DPmax 60 | GQ < 70 | AD > 2 | AB < 0.35 |
| **Wu et al. 2020** | GATK 3.7 - HaplotypeCaller and GenotypeGVCFs | QD < 2.0 ; MQ < 40.0; FS > 60.0; SOR > 3.0; MQRankSum< -12.5; ReadPosRankSum < -8.0 | pvalue poisson test 2 × 10^−4^ | GQ < 40 | AD = 0 at least for one parent | At least 3 reads with Alt and binomial test on allelic balance p-value < 0.05 |
| **Bergeron et al. 2021** | GATK 4.0.7.0 - HaplotypeCaller | QD < 2.0; MQ < 40.0; **FS > 20.0**; SOR > 3.0; **MQRankSum < -2.0; MQRankSum > 4.0; ReadPosRankSum < -3.0; ReadPosRankSum > 3.0** | DPmin 0.5 × av_t_ DPmax 2 × av_t_ | GQ < 60 | None | AB <0.3 AB > 0.7 |
| **Wang et al. 2021** | GATK version 3.6 | QD < 2.0; MQ < 40.0; FS > 60.0; **~~SOR > 3.0~~**; MQRankSum < -12.5; ReadPosRankSum < -8.0 | DPmin 20 DPmax 60 | GQ < 70 | AD > 0 | AB < 0.35 |

**Supplementary file 1d – PCR experiment and Sanger resequencing.** Primers for the 40 positions validated with PCR experiment. For each individual, the GenBank accession number are indicated as sequenceID with first the forward strand and then the reverse.

| **Chromosome** | **Position** | **Forward Primer sequence (5́-3́)** | **Reverse Primer sequence (5́-3́)** | **SequenceID father F/R** | **SequenceID mother F/R** | **SequenceID offspring F/R** | | **SequenceID 2nd offspring F/R** | |  |
| --- | --- | --- | --- | --- | --- | --- | --- | --- | --- | --- |
| chr2 | 5858286 | CTGAATTAGCCCACACTACA | CATGGAGTCACATTAAGGCT | EF31864958/- | -/EF31864936 | EF31877336/- | | EF31864919/EF31864921 | |  |
| chr2 | 150987129 | CATTAAGCCCCAGGTACATT | TTTTCCATAAATGGGCACCT | EF31873919/- | EF31872229/EF31872230 | EF31864006/EF31864007 | | EF31872228/EF31872315 | |  |
| chr2 | 203383064 | GGGGCATCTTCATTTTTCAC | ATACCCTATGCCACTTTACC | EF31873922/EF31873923 | -/EF31872242 | EF31877338/EF31877339 | | EF31872239/EF31872240 | |  |
| chr3 | 76549728 | TCTTAAGCCTCCACCAATTT | TTGTCAACATTGGTTCAACA | EF31873924/EF31873925 | EF31872253/EF31872254 | EF31864010/EF31864011 | | -/EF31872252 | |  |
| chr3 | 94315937 | TCACCCCACTTGTATTATGG |  | EF31864960/- | EF31864937/- | EF31864981/- | | EF31864922/- | |  |
| chr4 | 116799040 | GTTGTGGTCTGGGAGTATAA | GCTCCTACCTATTTGCTCTG | EF31873928/EF31873929 | EF31872277/EF31872278 | EF31864015/EF31864017 | | -/EF31872276 | |  |
| chr5 | 85980247 | GGAGACGCTTCTTATAACTCT | TACTGCTGCTCTCACTATC | EF31864961/EF31864962 | EF31864938/EF31864939 | EF31864593/EF31864594 | | EF31864924/EF31864925 | |  |
| chr5 | 118992462 | AAGCACTTGTAAGAGTACCCA | TCTCCCTGTAACATGACTTAAA | EF31873932/EF31873933 | EF31872303/EF31872304 | EF31864027/EF31864028 | | EF31872300/EF31872302 | |  |
| chr6 | 11241018 | CACAGGTAATAAAGAAGGGA | TTCCCACCAACAGTATAAAA | -/- | EF31872219/EF31872213 | EF31864093/EF31864094 | | EF31873848/EF31873857 | |  |
| chr6 | 18650255 | ATGCCAAGACTTGAGATGAG | GTCGTGACTGTGTTCTTAGT | EF31864581/EF31864582 | EF31872237/EF31872238 | EF31877378/EF31877379 | | EF31873904/EF31873914 | |  |
| chr6 | 64657185 | TCAAATTCATAGAGGCAGAA | ACAACCTGCTAAAAATGAAG | EF31864583/EF31864584 | EF31872249/EF31872250 | EF31877346/EF31877347 | | -/EF31873915 | |  |
| chr6 | 157547189 | CTTGTGTGGTATGCTTTATG | GATACCACAGATCTACAGAC | EF31873936/EF31873937 | EF31872231/EF31872232 | EF31864097/EF31864099 | | EF31873849/EF31873858 | |  |
| chr7 | 149216879 | CACAGTTGCTCTTCATTAGTC | CAGTCTGGAAACTCTCATCAT | EF31873938/EF31873939 | EF31872243/EF31872244 | | EF31877382/EF31877383 | | EF31873850/EF31873859 | |
| chr7 | 153210388 | CCACCAAAATTGCTCTTGAT | CAGCTTGTTCAGGGTTACTC | EF31873940/EF31873941 | EF31872255/EF31872256 | | EF31864033/EF31864034 | | EF31873851/EF31873860 | |
| chr8 | 7226159 and 7226160 | CGGTCATGTTAGGATGGAG | AAAGTACTTGAGTCCATGACA | EF31873942/EF31873943 | EF31872267/EF31872268 | | EF31877384/EF31877385 | | EF31873852/EF31873861 | |
| chr8 | 11458034 | GCTTTAATTAGCTTCAGTCCG | CATTAATTTTGACTGCCATGTG | EF31873944/EF31873945 | EF31872279/EF31872281 | | EF31877386/EF31877388 | | EF31873853/EF31873863 | |
| chr9 | 332616 | CACTCGATTAGCACCATTCT | GTGTTTCGAGCAATCTACAC | EF31873946/EF31873947 | EF31872292/- | | EF31864040/EF31864041 | | EF31873854/EF31873864 | |
| chr9 | 35992733 |  | TAAAGTCTTGCCCTCAGTTC | -/EF31864586 | -/EF31872262 | | -/EF31864043 | | -/EF31873916 | |
| chr9 | 36684503 | GAGCCATGTTCACGATTTTT (offspring:GATGTATGCTCTGAACAGGA) | AATGGCTGAATAGCATACCC | EF31864587/EF31864605 | EF31872273/EF31872274 | | EF31877391/EF31864046 | | EF31873908/- | |
| chr9 | 57966486 | CTCACTAAAATGAGGGGACA | TAGTTTTTCTCACACCCACAT | EF31873948/EF31873949 | EF31872305/EF31872306 | | EF31864047/EF31864048 | | EF31873855/EF31873865 | |
| chr9 | 59187032 | AGAAAGCAAGTGGTATGTGT | GTTGTAGTGTACAATCGCAT | EF31873950/EF31873952 | EF31872214/EF31872215 | | EF31864051/EF31864052 | | EF31873866/EF31873876 | |
| chr9 | 88707756 | TCTATATGACCCCAGGATAG | AGGATGTCTGTTATTACCAC | EF31873953/EF31873954 | EF31872233/EF31872234 | | EF31877393/EF31877394 | | EF31873867/EF31873877 | |
| chr9 | 93536046 | GGTCCCATTGGGGTAATATG | ATTGGTTGGCCTGAAGTTTA | EF31864546/EF31864547 | EF31872245/EF31872246 | | EF31877350/EF31877351 | | EF31873868/EF31873878 | |
| chr9 | 125284007 | GAGAGGGCTAATTTGGAAGT | ACAAGGAGGTATTTCGGTTG | EF31864548/EF31864603 | EF31872257/EF31872258 | | EF31877352/EF31877353 | | EF31873869/EF31873879 | |
| chr10 | 40351695 | ACATCTCCTTGACAGGATTG | TTGATGACCTGAGAAGTACC | EF31864550/EF31864551 | EF31872269/EF31872270 | | EF31877354/EF31877355 | | EF31873870/EF31873880 | |
| chr10 | 65456149 | GTTGGGTATGAATGTGGACT | GCTCAGAACGTTTTTATGGA | EF31864552/EF31864554 | EF31872282/EF31872283 | | EF31864066/EF31864067 | | -/EF31873881 | |
| chr11 | 44403758 | TACTGGGAACAGTACTGACA | ACTGGCTTGCTAAGGTTAAA | EF31864979/EF31864980 | EF31872294/EF31872295 | | EF31877356/EF31877357 | | EF31873872/EF31873882 | |
| chr11 | 79918225 |  | CCCAGCAATCTCTCTTCTTT | -/EF31873770 | -/EF31873769 | | -/EF31873744 | | -/EF31873768 | |
| chr11 | 98692026 | AGAGCTATAGGAAGGCGATT |  | EF31864589/- | EF31872286/- | | EF31864070/- | | EF31873909/- | |
| chr11 | 109301631 | TTTTAAAGACAAGGTCTCGC | TATTTGCCAAACGTCTATTG | EF31864557/EF31864559 | EF31872307/EF31872308 | | EF31864103/EF31864104 | | EF31873875/EF31873883 | |
| chr12 | 110983332 | TTTGGGGAGTGAGAGTAGAT | AACTGTCACATTCTCTCAGG | EF31864560/EF31864563 | EF31872216/EF31872224 | | EF31864072/EF31864073 | | EF31873884/EF31873893 | |
| chr13 | 28463207 | GTCAACACCTTAGAAAAGGC | TGTACTCTGTAAGGAGACGA | EF31864564/EF31864565 | EF31872235/EF31872236 | | EF31864074/EF31864075 | | EF31873885/EF31873895 | |
| chr13 | 79024526 | TATTGTAACACACAGGCCAA | GCTAAACGACATTTACCAGC | EF31864566/- | EF31872247/EF31872248 | | EF31877369/EF31877370 | | EF31873886/- | |
| chr13 | 105761009 | GCTAAAAACTCAATAAATTCGG | ATAAATTACCTTGGGCACTA | EF31864568/EF31864569 | EF31872259/EF31872260 | | EF31864105/EF31864106 | | EF31873887/EF31873898 | |
| chr14 | 41517101 | GTCTTAGGCAGTGATTGTGA | GAGCAAGAGCATCATTGTTT | EF31864570/- | EF31872271/EF31872272 | | EF31877401/EF31877402 | | EF31873888/EF31873899 | |
| chr14 | 52516157 | TTTGTGAGAACCCATCTCCG |  | EF31873773/- | EF31873772/- | | EF31873745/- | | EF31873771/- | |
| chr15 | 27373968 | GAAACACTGGAAGCACATAC | TTTGCACTCACTGATCAACT | EF31864573/EF31864574 | EF31872284/EF31872285 | | EF31864078/EF31864080 | | EF31873890/EF31873900 | |
| chr16 | 22611461 | AAGTCTTAATACATTGGGGG | GCAATATGGCAAGAGATTG | -/EF31864965 | -/EF31864944 | | EF31877407/EF31864988 | | EF31864927/- | |
| chr18 | 38858125 | GAATGATTCAAGGCTGTTCTC | ATGGCCACTAAAAATCACTT | EF31864604/EF31864578 | EF31872309/EF31872310 | | EF31877364/EF31877365 | | EF31873892/EF31873902 | |
